# Fructose uptake by brown adipose tissue is independent of carbohydrate response element-binding protein and does not cause elevated *de novo* lipogenesis: Fructose uptake by BAT is independent of ChREBP

**DOI:** 10.3724/abbs.2025229

**Published:** 2025-12-18

**Authors:** Janina Behrens, Marceline Manka Fuh, Daniel T. Haas, Michelle Y. Jaeckstein, Markus Heine, Bente Siebels, Anna Worthmann, Natalie Krahmer, Joerg Heeren, Ludger Scheja

**Affiliations:** 1 Department of Biochemistry and Molecular Cell Biology University Medical Center Hamburg-Eppendorf 20246 Hamburg Germany; 2 Institute for Diabetes and Obesity Helmholtz Diabetes Center Helmholtz Munich 85764 Neuherberg Germany; German Center for Diabetes Research (DZD) Helmholtz Munich 85764 Neuherberg Germany; 3 Section Mass Spectrometry and Proteomics University Medical Center Hamburg-Eppendorf 20246 Hamburg Germany

**Keywords:** brown adipose tissue, *de novo* lipogenesis, ChREBP, fructose, ceramides

## Abstract

Brown adipose tissue (BAT) is a heat-generating organ burning significant amounts of calories from fatty acids and glucose. The importance of glucose metabolism in the context of thermogenic function has been underlined by several studies. However, fructose metabolism and consequences of fructose overfeeding are poorly studied in BAT. Here we provide evidence that brown adipocytes use fructose as a substrate, however to a lesser extent than glucose. Furthermore, our data suggest that carbohydrate response element binding protein (ChREBP) and its target glucose transporter 5 (GLUT5) are not essential for fructose uptake and metabolism in BAT. Notably, we report that high fructose feeding has no effect on ChREBP activity and thus *de novo* fatty acid synthesis in BAT as opposed to liver and intestine. Instead, excessive carbohydrate loading of brown adipocytes induced by both, high-fructose feeding and impairment of ChREBP-dependent glucose metabolism, causes a massive accumulation of hexosylceramide species, as revealed by mass spectrometry-based lipidomics. Based on our data we hypothesize a reprogramming of fructose utilization upon impaired carbohydrate metabolism from canonical glycolysis and pentose phosphate pathway towards glycosphingolipid synthesis.

## Introduction

Fructose consumption in the form of added sugar in refined foods or sugar-sweetened drinks has strongly increased in many countries worldwide since the 1970s [ 1– 3] . Excessive fructose intake is associated with overweight and cardiometabolic disorders including hypertriglyceridemia, fatty liver disease, type 2 diabetes and cardiovascular diseases [ 3– 7] . The development of fatty liver and hypertriglyceridemia in response to fructose overfeeding is mechanistically well understood, in particular how it is linked to *de novo* lipogenesis (DNL), the metabolic pathway converting carbohydrates into long-chain fatty acids [8]. In human and rodent hepatocytes, fructose is rapidly metabolized to C _3_ intermediates of glycolysis via ketohexokinase (KHK), aldolase B (ALDOB) and triokinase (TKFC) in a relatively unregulated fashion [9], thereby providing acetyl-CoA as substrate for DNL. When in excess, fructose can thus promote liver steatosis and cause hypertriglyceridemia [ 9– 12] . In parallel to metabolically supplying the DNL pathway, fructose metabolism in hepatocytes efficiently stimulates the transcription of DNL enzyme genes, predominantly through activation of the transcription factor carbohydrate response element-binding protein (ChREBP) [ 9, 13] . As shown by studies in liver-specific *ChREBP*-knockout mice, activation of ChREBP is essential for the development of obesity and disturbed glucose homeostasis in mice fed with a high-fructose diet [14]. Several members of the hexose transporter family, SLC2A2 (GLUT2), SLC2A5 (GLUT5) and SLC2A8 (GLUT8), have been implicated in hepatocyte fructose uptake [ 2, 9, 15] . Of note, the selective fructose transporter SLC2A5 [16] is activated by ChREBP in both the small intestine and the liver, a regulation that establishes a feed-forward mechanism linking fructose uptake, fructose metabolism and DNL [ 14, 17] . Quantitative postprandial studies have determined that a significant proportion of fructose is metabolized already in enterocytes of the small intestine and converted into glucose via gluconeogenesis [18]. In addition, microbiota in the colon convert fructose into short-chain fatty acids [ 18– 20] . Intestinal metabolism may be the primary route for ingested fructose when moderate amounts of the sugar are consumed, at least in mice [ 9, 18] . If fructose intake is high, a larger portion of the monosaccharide is directed to the liver via the portal vein and is cleared by hepatocytes [ 11, 18] . In consequence of the splanchnic processing, only a minor proportion of ingested fructose reaches the systemic circulation [21] and the concentration of fructose in peripheral blood is low compared to glucose [ 21– 23] . Nevertheless, systemic fructose concentrations rise postprandially [21] and fructose use in peripheral organs can be of relevance. For example, fructose metabolism has been shown to promote the development of cardiomyopathy in mice [24]. The significance of fructose utilization in adipose tissue as another major site of energy metabolism is not well researched. *Slc2a5* is expressed in white adipocytes [25] and *Slc2a5*-KO mice have reduced epididymal white fat mass [26]. In line with this observation, fructose promotes the differentiation of 3T3-L1 preadipocytes [ 26, 27] .

Even less is known about the functional significance of fructose metabolism in brown adipose tissue (BAT). BAT is a heat-generating (thermogenic) organ unique to placental mammals that is designed to counteract drops in body temperature in response to a cold surrounding [28]. In such a context, BAT is activated by increased sympathetic tone and subsequently takes up large amounts of energy substrates from the circulation including glucose, fatty acids and others [ 29– 33] , next to releasing fatty acids from intracellular lipid droplets [ 28, 34] . The abundant energy generated from oxidation of these molecules is converted to heat through uncoupling protein 1 (UCP1), a protein that uncouples the electron transfer chain from ATP synthesis by shuttling protons through the inner mitochondrial membrane [ 28, 35, 36] . Of note, a portion of glucose taken up by activated brown adipocytes is not completely oxidized to CO _2_ but is initially converted to fatty acids by DNL [37], which is very prominent in cold-activated BAT [ 37– 39] . The metabolic by-pass of glucose through fatty acids is not well understood but might contribute to thermogenesis independent of UCP1 by metabolic futile cycling or it may play more indirect roles such as preventing tricarboxylic acid cycle overload [40]. Overall, to what extent fructose is used for DNL in BAT is not known.

In the current study, we aimed to investigate, whether BAT uses fructose as a source for energy and how this organ responds to fructose overfeeding. Beyond that, we analyzed how the lack of ChREBP, the dominant transcription factor for DNL in BAT [39], affects fructose handling. Conversely, we determined how ChREBP activity in brown adipocytes is affected by dietary fructose. Surprisingly, we found that *Slc2a5* expression in BAT is highly dependent on ChREBP but that SLC2A5 is not essential for fructose uptake into BAT. Furthermore, we found that high fructose feeding does not modulate DNL in BAT as it does in liver. Unexpectedly, dietary fructose together with ChREBP deficiency results in massive accumulation specifically in BAT of hexosylceramides (HexCer), glycosphingolipids mainly located in plasma membranes [ 41– 43] that are the starting points for the synthesis of GM3 gangliosides and other complex glycolipids [44] implicated in cellular signaling and the development of insulin resistance [ 45, 46] . Taken together, we discovered HexCer synthesis as an alternative metabolic fate of excessive carbohydrates in murine brown adipocytes, which has potential implications in the context of over-nutrition-related cardiometabolic diseases.

## Methods and Materials

### Mice

Mouse studies were approved by the Animal Welfare Officers of University Medical Center Hamburg-Eppendorf (UKE) and Behörde für Gesundheit und Verbraucherschutz Hamburg. Unless indicated otherwise, female mice were used. Age- (11 to 15 weeks) and weight-matched wild-type C57BL/6 mice (purchased from Janvier, Le Genest-Saint-Isle, France), global *ChREBP*-knockout [39], ChREBPα ^fl/fl^ Ucp1 Cre-positive (Cre ^+^) mice [39] and control littermates (Cre ^–^), generated by crossing Ucp1-Cre ^Evdr^ mice [47] with ChREBPα ^fl/fl^ mice [48], were housed at room temperature (22°C) or at cold (6°C) for 7 days at a 12 h light/12 h dark cycle with *ad libitum* access to water and food (if not stated otherwise chow diet; Altromin, Lage, Germany). Importantly, the ChREBPα ^fl/fl^ Ucp1 Cre ^+^ mice exhibit loss of ChREBPα in brown adipocytes due to deletion of exon1a but also lack the other ChREBP isoform, ChREBPβ, because its expression is ChREBPα-dependent [49]. For organ harvest, mice were anesthetized with ketamine (180 mg/kg)/ xylazine (24 mg/kg), and systemically perfused with PBS via the left heart ventricle. Organs were harvested and snap-frozen in liquid nitrogen.

### High fructose diet feeding

ChREBPα ^fl/fl^ Ucp1 Cre ^+^ mice and control littermates were fed with chow diet until the age of 11 to 12 weeks. Afterwards, mice of each genotype were continued to be fed with chow diet and four mice of each genotype were fed with the 60% fructose diet Teklad Custom Diet from Envigo/Inotiv (TD.89247; West Lafayette, USA) for one week. The mice were housed at 22°C throughout the study.

### Cells

Isolation of primary brown adipocytes was adapted from a protocol by Fischer and Jaeckstein [50]. In brief, BAT was minced with surgical scissors and digested for 30 min at 37°C in digestion buffer (PBS, 10 mM CaCl _2_, 2.4 units/mL dispase II, and 1.5 units/mL collagenase D). Homogenate was passed through a 100-μm cell strainer and flow-through was incubated on ice for 30 min to separate preadipocytes and mature adipocytes. Lower phase (2/3 of the volume) was passed through a 40-μm cell strainer and the flow-through was centrifuged for 10 min at 800 *g*. The pellet was resuspended in differentiation media [DMEM/high glucose supplemented with 10% new-born calf serum (NCS), 1% penicillin/streptomycin, 1% antibiotic/antimycotic, 2.4 nM insulin and 1 μM rosiglitazone]. Cells were differentiated for 7 days with daily media change. By using this established brown adipocyte protocol, a differentiation rate of 80%–90% was achieved. On day 6, cells were changed to glucose free medium (DMEM with 10% NCS) and supplemented with 25 mM glucose or 1 mM fructose for 24 h. Cells were washed twice with ice cold PBS and harvested in TRIzol reagent (Thermo Fisher Scientific, Waltham, USA) for RNA isolation.

### Metabolic tracer studies

Mice were fasted for 4 h. Afterwards they were injected intravenously with ^14^C-labeled fructose (0.36 MBq/kg body weight) and ^3^H-labeled deoxyglucose (0.72 MBq/kg body weight). After 10 min, mice were anesthetized with ketamine (180 mg/kg)/xylazine (24 mg/kg), and systemically perfused with PBS-heparin (10 U/mL) via the left heart ventricle. Organs were harvested and lysed for counting using Solvable and counted in a TriCarb scintillation counter (Perkin Elmer, Waltham, USA).

Radioactive tracer uptake study with primary brown adipocytes was conducted on day 7 of differentiation. Cells were washed twice and incubated for 2 h with DMEM with 1 g/L glucose without serum. Afterwards, cells were incubated with ^14^C-labeled fructose (0.0037 MBq/μL) and ^3^H-labeled desoxyglucose (0.037 MBq/μL) for 60 min. Cells were washed twice, lysed with 0.1 M NaOH and counted in a TriCarb scintillation counter (Perkin Elmer). Protein concentrations were determined by BCA assay (23225; Thermo Fisher Scientific). The amount of tracer, which was taken up by the cells, was calculated based on the radioactive decay per minute and the specific activity of the tracer.

### Magnetic activated cell sorting

The cell sorting method was adapted from Fischer and Jaeckstein [50]. In brief, BAT (pool of 4) were minced with surgical scissors, and digested for 45 min at 37°C in PBS containing 10 mM CaCl _2_, 2.4 units/mL dispase II, and 1.5 units/mL collagenase D. After digestion, the homogenate was passed through a cell strainer (100 μm), and the flow-through was centrifuged for 5 min, at 600 *g*, at 4°C. The pellet was resuspended in PBS containing 2 mM EDTA, 0.5% BSA, 2 mM glucose, and filtered through a cell strainer (40 μm). The filtrate was centrifuged at 600 *g* for 5 min, the cell pellet was resuspended and incubated with CD11b MicroBeads (10 μL beads/10 ^7^ cells; Miltenyi Biotec, Bergisch Gladbach, Germany) for isolation of the monocyte fraction. CD11b ^+^ cells were captured from the lysate using magnetic columns (Miltenyi Biotec). The flow-through was centrifuged, the pellet was resuspended and incubated with CD31 MicroBeads (10 μL beads/10 ^7^ cells; Miltenyi Biotec) to isolate endothelial cells. The flow-through, containing preadipocytes, was collected. Cell fraction pellets were dissolved in TRIzol reagent for RNA extraction.

### Gene expression analysis

Tissues or cells were homogenized (20 Hz for 2 × 3 min) in 1 mL of TRIzol reagent using a TissueLyser type 3 (QIAGEN, Hilden, Germany). A total of 250 μL chloroform was added, samples were mixed and centrifuged. Supernatant was added to 600 μL of 70% ethanol. Further purification was performed by using NucleoSpin RNAII Kit (Machery&Nagel, Dueren, Germany) according to the manufacturer’s instructions. Double-stranded DNA was digested using rDNase I. Purified RNAs (400 ng) were used for cDNA synthesis using High-Capacity cDNA Archive Kit (Thermo Fisher Scientific) and reverse transcription PCR program was as followed: (1) 10 min, 25°C; (2) 120 min, 37°C; 3.5 s, 85°C. Gene expression was assessed using Taqman assays supplied by Thermo Fisher Scientific or self-designed SYBR primer.

Assays on demand: Tbp (00446973_m1), Mlxipl_exon1a-2 = Mlxipl isoform 1 (Chrebpα) (01196407_m1), Mlxipl_exon1b-2 = Mlxipl isoform 2 (Chrebpβ) (AIVI4CH), Fasn (00662319_m1), Acaca (Mm01304285_m), Slc2a5 (Mm00600311_m1), Slc2a8 (Mm00444634_m1), Slc2a9 (Mm00455122_m1), Slc2a12 (Mm02375931_s1), Ugcg (Mm00495925_m1), Ugt8a (Mm00495930_m1), St3gal5 (Mm00488237_m1), Gba (Mm00484700_m1), Galc (Mm00484646_m1), Slc2a1 (Mm00441480_m1), Slc2a2 (Mm00446224_m1) Slc2a4 (Mm01245502_m1), Hk2 (Mm00443385_m1), Khk (Mm00434647_m1), Gpi1 (Mm01962484_u1), Pkm (Mm00834102_gH), Gpd1 (Mm00515846_m1), Gpd2 (Mm00439082_m1), Gapdh (Mm99999915_g1), Aldoa (Mm00833172_g1), and Aldob (Mm00523293_m1)

SYBR-primer: *Taldo1* (Fw. 5′-GAAGACCAAATGGCCGTGGA-3′, Rv. 5′-ACTACTTCCCGTTCTCAGCG-3′), *G6pd* (Fw. 5′-GGAGAAGGAGAAGCTGCCAA-3′, Rv. 5′-ATGAAGGGTACCCCATCCCA-3′), *Pgls* (Fw. 5′-GGCCATACCTGTTCGCTCTT-3′, Rv. 5′-TCAGCACAGGAAGCGTTAGG-3′), *Tkt* Fw. 5′-GGGCTGGTGTAACTCTGCAT-3′, Rv. 5′-TCCTGTCCAGGGGCTTGATA-3′), *Pgd* (Fw. 5′-GCAAGTTCTGGGCTACCCTT-3′, Rv. 5′-CCGTTGTGCACCATCTTCAC-3′), *Khk-a* (Fw. 5′-TTGCCGATTTTGTCCTGGAT-3′, Rv. 5′-CCTCGGTCTGAAGGACCACAT-3′, these primer specifically detect exon 3a) [51], *Khk-c* (Fw. 5′-AACTCCTGCACTGTCCTTTCCTT-3′, Rv. 5′-CCACCAGGAAGTCGGCAA-3′, these primer specifically detect exon 3c) [51]

For mRNA sequencing (bulk RNA-seq), total purified RNA from BAT of wild type and global *ChREBP*-knockout mice was sent to Novogene (Cambridge, UK). The company performed quality check, library construction and transcriptome sequencing on a NovaSeq 6000 PE150 platform. The bioinformatics analysis included mapping to the mouse reference genome, gene expression quantification, differential expression analysis and gene ontology (GO) enrichment analysis (including three main branches; cellular component, molecular function, biological process). GO terms with padj < 0.05 were considered significantly enrichment.

### Western blot analysis

Tissues were homogenized (20 Hz for 2 × 3 min) in 10× (v/w) RIPA buffer (50 mM Tris-HCl pH 7.4; 5 mM EDTA; 150 mM NaCl; 1 mM Na-pyrophosphate; 1 mM NaF; 1 mM Na-vanadate; 1% NP-40) supplemented with complete Mini protease inhibitor (Roche, Basel, Switzerland) using TissueLyser-type3 (QIAGEN). Samples were centrifuged and supernatant was collected without upper lipid layer contamination. Protein was quantified by BCA assay. Sample concentration was adjusted with RIPA buffer and 2-fold NuPAGE® LDS Sample buffer + Sample Reducing Agent (Invitrogen) was added. Total proteins (20 μg) were separated by 10% Tris-glycine SDS-PAGE and transfered to nitrocellulose membranes (GE Healthcare, Waukesha, USA) in a wet blotting system [blotting buffer: 20 mM Tris, 150 mM glycine, 20% (v/v) methanol] overnight at 200 mA. Membranes were stained with Ponceau Red (Sigma, St Louis, USA), cut and blocked for 1 h in 5% milk in TBS-T [20 mM Tris, 150 mM NaCl, 0.1% (v/v) Tween 20]. Membranes were incubated overnight at 4°C with the corresponding primary antibodies diluted 1:1000 in 5% BSA (Sigma) in TBS-T. After washing in TBS-T, membranes were incubated for 1 h at room temperature with the corresponding HRP-conjugated secondary antibody diluted 1:5000 in 5% milk in TBS-T. After washing in TBS-T, detection was performed with Amersham Imager 600 (GE Healthcare) using SuperSignal West Femto ECL (Thermo Fisher Scientific). Primary antibodies are shown as: UCP1 (#MAB6158; R&D Systems, Minneapolis, USA), ACC (#3662; Cell Signaling Technologies, Danvers, USA), FASN (#610962; BD Biosciences, Heidelberg, Germany), γTUB (#ab179503; Abcam, Cambridge, UK). HRP-conjugated secondary antibodies: α-mouse (#115-035-146; Jackson Immunoresearch, West Grove, USA) and α-rabbit (#111-03-144; Jackson Immunoresearch).

### Proteomic sample preparation

Tissue sections were lysed in lysis buffer (2% sodium deoxycholate, 100 mM Tris, pH 8.5) using a glass homogenizer. Samples were boiled at 95°C for 5 min at 1000 rpm in a thermoshaker and subsequently sonicated using a Diagenode Bioruptor (Diagnode, Liege, Belgium; 15 cycles of 30 s). Protein concentrations were determined by BCA assay, and 50 μg of total protein per sample were adjusted to a final volume of 100 μL. Reduction was performed with 10 mM Tris(2-carboxyethyl)phosphine (TCEP) and alkylation with 40 mM chloroacetamide (CAA) at 45°C for 10 min at 1000 rpm in the dark. Proteins were digested overnight at 37°C and 1000 rpm using trypsin (T6567; Sigma) and LysC (129-02541; FUJIFILM Wako, Neuss, Germany) in a 1:50 enzyme-to-protein ratio. Samples were acidified by adding an equal volume of 2% trifluoroacetic acid (TFA) in isopropanol, centrifuged at 15,000 *g* for 10 min, and the supernatant containing peptides was collected. Peptide desalting was performed using in-house packed StageTips with three layers of styrenedivinylbenzene reversed phase sulfonate (SDBRPS, 3M Empore; Merk, Darmstadt, Germany). StageTips were washed with 100 μL acetonitrile (ACN), activated with 100 μL of 30% methanol containing 1% TFA, and equilibrated with 150 μL 0.2% TFA. All centrifugation steps were performed at 1000 *g* for 5 min or until complete flow through. Peptides were eluted with 60 μL of elution buffer (80% ACN, 1.25% NH _4_OH), lyophilized, and resolved in 6 μL MS loading buffer (2% CAN, 0.1% TFA). Peptide concentrations were measured using a nanophotometer (Implen N60; Implen GmbH, Munich, Germany).

### Proteomic acquisition

LC-MS/MS analysis was performed on an Orbitrap Exploris 480 mass spectrometer (Thermo Fisher Scientific) equipped with an electrospray ionization (ESI) source and a High-Field Asymmetric Waveform Ion Mobility Spectrometry System (FAIMS; Thermo Fisher Scientific) interface, coupled to an EASY-nLC 1200 HPLC system (Thermo Fisher Scientific). Peptide separation was conducted at 60°C using an in-house packed 50-cm analytical column (75 μm inner diameter) containing ReproSil-Pur C18-AQ 1.9 μm resin (Dr. Maisch). A 60-min gradient was applied using a binary solvent system of buffer A (0.1% formic acid) and buffer B (80% ACN, 0.1% formic acid), with the following profile: 5%–20% buffer B over 30 min, 20%–29% buffer B over 9 min, 29%–45% buffer B over 6 min, 45%–95% buffer B over 5 min, held at 95% for 5 min, and re-equilibrated to 5% over 5 min at a constant flow rate of 300 nL/min. Data Independent Acquisition (DIA) was performed using 33 variable-width isolation windows. FAIMS was operated at a compensation voltage of -50 V. ESI voltage was set to 2650 V, and the temperature of the ion transfer tube was set to 275°C. For MS1 scans, the Orbitrap resolution was set to 120,000 with a scan range of 300–1650 m/z, normalized AGC target of 300%, and a maximum injection time of 45 ms. MS2 scans were acquired at a resolution of 15,000, with a first mass of 120 m/z (window dependent), normalized AGC target of 1000%, and maximum injection time of 22 ms. All measurements were performed in positive ion mode.

### Proteomic data processing

Raw MS data were analyzed using Spectronaut version 18 (Biognosys) with the directDIA+ workflow in deep mode. Spectral searches were performed against the Mus musculus UniProt reference list (UP000000589_10090), allowing for Trypsin/P cleavage specificity, peptide lengths of 7–52 amino acids, and up to two missed cleavages. Carbamidomethylation (C) was set as fixed modification, while methionine oxidation and N-terminal acetylation were included as variable modification. Quantification based on MaxLFQ with 3-6 best N fragment ions per peptide.

### Plasma analysis

Plasma was obtained from heart blood by addition of EDTA and centrifugation. Plasma glucose levels were measured using an Accu-Check Guide (Roche). For photometric determination of plasma triglycerides and cholesterol, 5 μL plasma was used. A total of 200 μL of the respectively reaction regent (triglyceride/cholesterol kit; DiaSys, Holzheim, Germany) were added and the mixture was incubated at 37°C for 10 min. Extinction at 500 nm was measured using an ELISA-Reader (Multiscan GO; Thermo Fisher Scientific). For photometric determination of plasma fructose, 10 μL of plasma was used. A sample blank was performed by adding 200 μL of the glucose assay reagent (#FA-20; Sigma) to the sample, which was followed by 15 min of incubation at room temperature. Absorbance was measured at 340 nm. For fructose determination, 2 μL phosphoglucose isomerase (PGI) was added to convert fructose into glucose. Then 200 μL of the glucose assay reagent was added and samples were incubated for 15 min at room temperature. Absorbance was measured at 340 nm and sample blank was subtracted. Concentrations were calculated based on fructose standard. Total ketone body concentrations in plasma were determined by the Autokit Total Ketone Bodies from FUJIFILM (FUJIFILM Wako). In brief, 4 μL plasma or standard was incubated with 270 μL R1 regent for 5 min at 37°C. Afterwards, 90 μL of R2 reagent was added and extinction (E) at 405 nm was measured every 30 s for 5 min. Delta E between minute 1 and minute 2.5 was used to calculate ketone body concentrations.

Insulin concentrations in plasma samples were determined using the Ultra Sensitive Rat Insulin ELISA Kit (Cat. No. 90060; CrystalChem, Downers Grove, USA). Insulin standard or sample (5 μL) was added to 95 μL of dilution buffer into the pre-coated wells and incubated at 4°C for 2 h. Afterwards, wells were washed 5 times with 1-fold washing buffer. Anti-insulin enzyme conjugat (100 μL) was added and plate was incubated for 30 min at room temperature. Washing procedure was repeated. Enzyme substrate solution (100 μL) was added and wells were incubated for 40 min at room temperature protected from light. After addition of stop solution, absorbance at 450 nm and 630 nm was measured using an ELISA-Reader. A630 was subtracted from A450 and insulin concentrations were calculated based on standard curve.

### Haematoxylin and eosin stain (HE stain)

HE stain was performed on paraffin embedded tissues (BAT). Sections (5 μm) were cut on a Leica Microtome and mounted on Histobond slides (Marienfeld-Superior). Deparaffination was performed by xylol substitute incubation and sections were dehydrated by descending ethanol chain. Slides were incubated in haematoxylin solution for 15 min, rinsed under water and incubated for 1 min in eosin. Slides were mounted using Eukitt.

### Lipidomics

Lipidomic analysis was performed as described by Su and Worthmann [ 52, 53] . Briefly, after addition of internal standards, lipids were extracted from 3 mg of snap frozen iBAT using an adjusted MTBE/methanol extraction protocol [54]. Lipid extracts were concentrated and reconstituted in running buffer [10 mM ammonium actetate, dichloromethane (50): methanol (50)] and analyzed by a flow injection analysis-tandem mass spectrometry (FIA-MS/MS)-based method using an ultra-high pressure liquid chromatography system (Nexera X2; Shimadzu, Kyoto, Japan) coupled with a QTRAP system (QTRAP 5500; AB SCIEX Germany, Darmstadt, Germany) run in multiple-reaction monitoring mode and equipped with a differential mobility spectrometer (DMS) interface operating with SelexION technology. Acquired raw data was processed and lipids were quantified using the Shotgun Lipidomics Assistant, a Python-based application.

### Statistical analysis

The statistical tests used can be found in the figure legends. In general, two groups were compared by unpaired two-tailed Student’s *t* test, more than two groups by one-way ANOVA. Statistical analyses were conducted using Graph Pad Prism software; *P*  < 0.05 was considered statistically significant.

## Results

### The ChREBP target SLC2a5 is not an essential fructose transporter in brown adipose tissue

**Figure 1:**
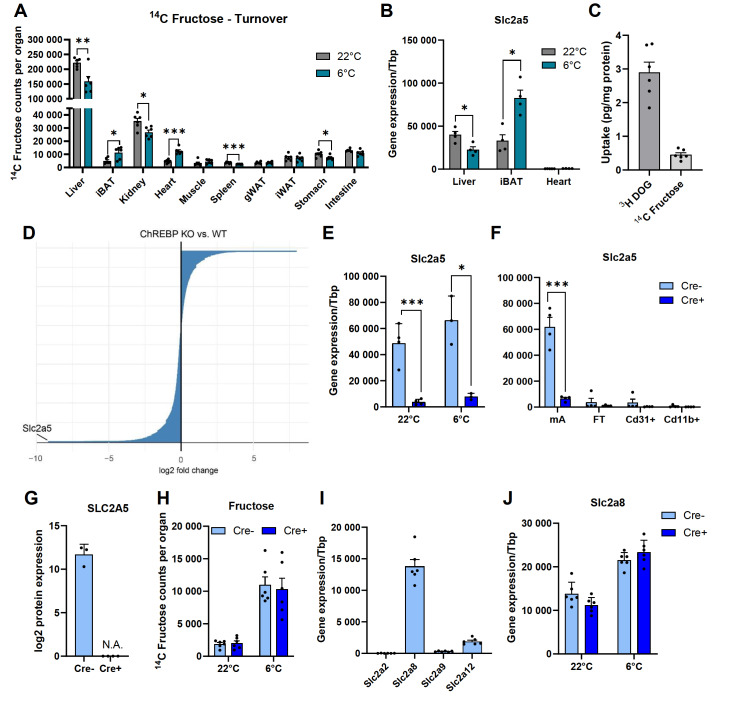
Figure 1 The ChREBP target Slc2a5 (GLUT5) is not an essential fructose transporter in brown adipose tissue (A) 14C fructose counts after intravenous administration measured in organs of male wild-type mice housed at 22°C or 6°C for 7 days. (B) Gene expression of Slc2a5 relative to Tbp (housekeeping gene) measured in liver, iBAT and heart of wild-type mice housed at 22°C or 6°C for 7 days. (C) Uptake of 3H-DOG and 14C-fructose into differentiated primary brown adipocytes. Incubation time was one hour. (D) mRNA-seq data from BAT of wild-type controls and global ChREBP-KO mice housed at 22°C. Shown are log2 fold change ChREBP-KO vs wild-type (WT), n = 4. (E) Gene expression of Slc2a5 relative to Tbp measured in BAT of ChREBPαfl/fl Ucp1 Cre+ mice and Cre– controls housed at 22°C or 6°C. (F) Gene expression of Slc2a5 relative to Tbp measured in MACS fractions [mature adipocytes (mA), flow-through (FT), CD31-positive cells (Cd31+), CD11b-positive cells (Cd11b+)] of BAT from male ChREBPαfl/fl Ucp1 Cre+ mice and Cre- controls housed at 22°C. Three BATs per pool, n = 4 pools. (G) SLC2A5 protein level (MS-based proteomics approach) in male ChREBPαfl/fl Ucp1 Cre+ mice and Cre– controls housed at 6°C. (H) 14C fructose counts 10 min after intravenous administration measured in BAT from ChREBPαfl/fl Ucp1 Cre+ mice and Cre– controls housed at 22°C or 6°C for 7 days prior to injection. (I) Gene expression of Slc2a isoforms in BAT of Cre– mice housed at 22°C. (J) Gene expression of Slc2a8 relative to Tbp measured in BAT of ChREBPαfl/fl Ucp1 Cre+ mice and Cre- controls housed at 22°C and 6°C for 7 days. (A–C,E–J) Data are presented as the mean ± SEM, Student`s t-test. *P < 0.05, **P < 0.01, ****P < 0.001.

BAT is a metabolic sink with a high ability to take up various energy substrates including glucose, amino acids and lipids from the circulation and use them for thermogenesis [ 29– 33] . Fructose has not been studied in this context, which prompted us to determine organ fructose uptake in a turnover experiment. We used the intravenous route because the majority of ingested fructose is converted to glucose by enterocytes [18], which makes interpretation of orally applied fructose tracer difficult. Mice were housed at room temperature (22°C) or adapted to cold (6°C) for 7 days to achieve conditions of moderate and high BAT thermogenic capacity, respectively. In the current study, we applied a 7-day chronic cold exposure regimen to investigate cold-adapted BAT that has undergone tissue remodeling and is in a stable state [55]. ^14^C-labeled fructose and ^3^H-labeled deoxyglucose ( ^3^H-DOG) as a control for glucose uptake were intravenously administered and organs were harvested 10 min later ( Figure 1A). As expected, the majority of fructose was taken up by the liver, consistent with its prominent role in fructose clearance [ 11, 18] . Fructose uptake into BAT was much lower than in liver and similar to that in the heart and white adipose tissue (WAT) depots at room temperature ( Figure 1A). Notably, in cold-adapted mice, the quantity of fructose taken up by BAT and heart was more than twice as high as in mice housed at room temperature ( Figure 1A). Conversely, the liver, but also kidneys, stomach and spleen showed reduced fructose uptake under cold exposure ( Figure 1A). Tracking with the reciprocal regulation of fructose uptake, the expression of the fructose transporter *Slc2a5* was higher in BAT but lower in liver of cold-adapted versus control-housed mice, whereas heart showed negligible *Slc2a5* mRNA ( Figure 1B). These findings indicate that after cold exposure, fructose is redistributed to BAT and heart. A similar organ distribution pattern was observed for ^3^H-DOG ( Supplementary Figure S1A), a measure of glucose transport. To estimate the ratio of fructose taken up by brown adipocytes in comparison to glucose, we conducted an uptake experiment with primary brown adipocytes again using ^3^H-DOG and ^14^C fructose as tracers. Fructose was taken up by the cells, however, at lower amounts compared to DOG ( Figure 1C). To explore whether the transcription factor ChREBP controls *Slc2a5* in BAT as it does in other organs [14], we analyzed bulk mRNA-seq data with BAT from global *ChREBP*-knockout and wild-type control mice housed at 22°C. Notably, *Slc2a5* was the most downregulated gene in ChREBP-deficient BAT ( Figure 1D), indicating that *Slc2a5* is also a ChREBP target in BAT. This notion was confirmed in BAT-specific *ChREBPα*-knockout mice with a floxed exon1a and Cre recombinase driven from the *Ucp1* promoter. The mouse model is characterised by significant reduction of DNL, as shown by reduced content of the DNL-derived fatty acids lauric acid (12:0), myristic acid (14:0), myristoleic acid (14:1), palmitic acid (16:0) and palmitoleic acid (16:1) in BAT triglycerides ( Supplementary Figure S1B). The ChREBPα ^fl/fl^ Ucp1 Cre ^+^ mice exhibited strongly reduced *Slc2a5* compared to Cre ^–^ controls under both housing conditions, room temperature and chronic cold exposure ( Figure 1E). As shown by magnetic activated cell-sorting of collagenase-digested BAT, *Slc2a5* was highly expressed in mature brown adipocytes but less so in CD11b ^+^ myeloid cells, CD31 ^+^ endothelial cells and in the flow-through fraction that contains the remaining cell types ( Figure 1F). Importantly, the knockout was also manifest at protein level, as no SLC2A5 was detected by mass spectrometry-based proteomics in BAT of Cre ^+^ mice ( Figure 1G). Together, these data suggested a potential role of ChREBP and SLC2A5 in controlling fructose uptake and the response to cold in BAT. However, in turnover experiments using radiolabelled fructose administered intravenously, the clearance of fructose by BAT was similar in Cre ^+^ and Cre ^–^ mice. This was observed at both 22°C and 6°C ( Figure 1H). Based on this result we concluded that *Slc2a5* is not an essential fructose transporter in BAT. To elucidate which other transporter described to shuttle fructose [56] might be important for BAT, we determined gene expression levels of *Slc2a2*, *Slc2a8*, *Slc2a9* and *Slc2a12* in BAT and primary brown adipocytes. Of note, *Slc2a8* described to play a crucial role for fructose uptake into the liver and intestine [ 15, 57] , was by far the most highly expressed transporter ( Figure 1I and Supplementary Figure S1C). Other proposed fructose transporters, *Slc2a9* and *Slc2a12*, also displayed reasonable expression ( Figure 1I). Of note, among these, *Slc2a8* expression showed the strongest correlation with BAT fructose uptake ( Supplementary Figure S1D–F) and was independent of ChREBP ( Figure 1J). Taken together, we find that fructose is taken up into brown adipocytes and that the conventional fructose transporter SLC2A5 and its key regulator ChREBP are not critical for this process.

### High-fructose feeding does not enhance DNL in BAT

**Figure 2:**
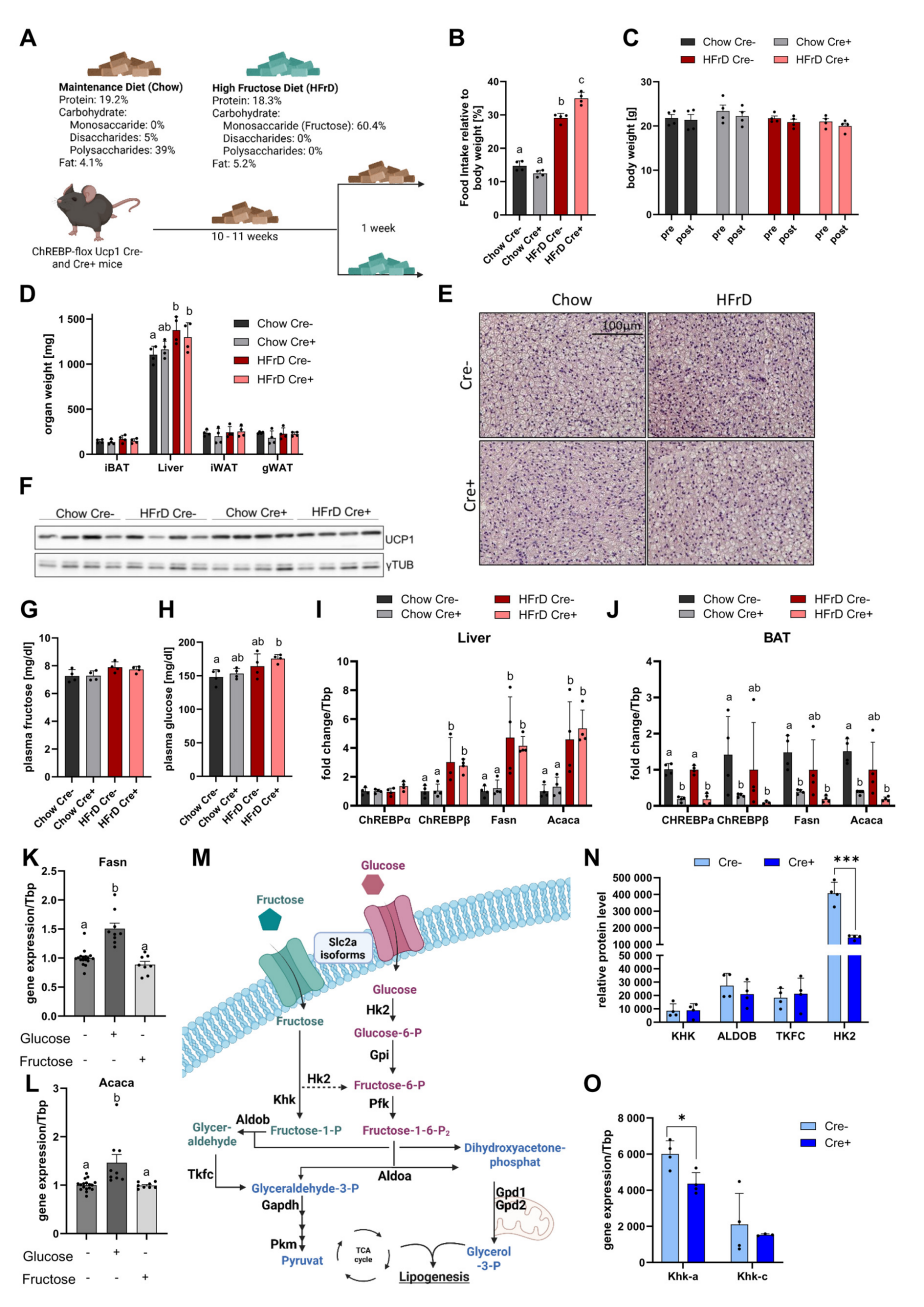
Figure 2 High-fructose feeding does not enhance DNL in BAT (A) Experimental setup and diet composition of the feeding study. (B) Food intake by weight, (C) body weight and (D) organ weight of ChREBPαfl/fl Ucp1 Cre+ mice and Cre– controls fed with control (chow) or high fructose (HFrD) diet. (E) Haematoxylin eosin staining of BAT samples from ChREBPαfl/fl Ucp1 Cre+ mice and Cre– controls fed with chow or high fructose diet. Bar corresponds to 100 μm. Representative images of n = 4 mice, with n = 3 images per mouse are shown. (F) Western blot analysis of UCP1 from iBAT of ChREBPαfl/fl Ucp1 Cre+ mice and Cre– controls fed with chow or high fructose diet. Plasma (G) fructose and (H) glucose of ChREBPαfl/fl Ucp1 Cre+ mice and Cre– controls fed with chow or high fructose diet. Fold change gene expressions of ChREBPα, ChREBPβ, Fasn and Acaca in (I) liver and (J) BAT of ChREBPαfl/fl Ucp1 Cre+ mice and Cre– controls fed with chow or high fructose diet. Gene expression of (K) Fasn and (L) Acaca in primary brown adipocytes supplemented with 25 mM glucose or 1 mM fructose. Data from n = 3–4 independent experiments. (M) Schematic overview of carbohydrate metabolism. (N) Relative protein expression of KHK, ALDOB, TKFC and HK2 (MS-based proteomics approach) in male ChREBPαfl/fl Ucp1 Cre+ mice and Cre– controls housed at 22°C. (O) Gene expression of the Khk-a and Khk-c splice variants in BAT of ChREBPαfl/fl Ucp1 Cre+ mice and Cre– controls housed at 22°C. Data are presented as the mean ± SEM. (B–D,G–L) one-way-Anova, P < 0.05 is considered significant, different letters denote significant differences; (N,O) Student’s t-test, *P < 0.05, ***P < 0.001.

Previous studies revealed that fructose feeding stimulated ChREBP activity in enterocytes and hepatocytes [ 14, 17] . To test whether this is also the case in BAT and whether this is directly dependent on ChREBP activity, we fed BAT-specific *ChREBPα*-knockout mice and Cre ^–^ control mice for one week with a high fructose diet (HFrD) frequently used to study fructose overfeeding [ 58– 60] ( Figure 2A). Compared to chow diet-fed mice, food intake in mice receiving HFrD was nearly doubled ( Figure 2B), but this had no effect on body weight ( Figure 2C). Liver weight was, however, slightly increased after HFrD ( Figure 2D), an effect that has been reported before and reflects liver steatosis [ 61– 63] . BAT and WAT depots showed no effect of HFrD feeding ( Figure 2D). Moreover, BAT morphology and lipid droplet appearance ( Figure 2E) as well as UCP1 protein level ( Figure 2F and Supplementary Figure S2A) were unchanged after HFrD feeding. Plasma fructose and glucose level tended to be higher after one week of HFrD in Cre ^–^ and Cre ^+^ mice ( Figure 2G,H) but no differences between the genotypes and diets were detected for triglycerides, cholesterol and insulin ( Supplementary Figure S2B–D). Of note, plasma ketone body levels were strongly reduced upon HFrD feeding in both genotypes ( Supplementary Figure S2E), which is in line with a previous study showing an anti-ketogenic effect of fructose [64].

Next, we addressed whether fructose feeding induces DNL gene expression. Consistent with previous publications, we observed increased expression of the DNL enzymes *Acaca* and *Fasn* in the liver. Meanwhile, hepatic mRNA expression of the ChREBP isoform ChREBPβ, a surrogate marker of ChREBP activity [49], was elevated ( Figure 2I). As expected from the BAT-specific knockout, ChREBP and DNL enzyme expression was strongly reduced in BAT but not influenced by genotype in liver ( Figures 2I,J). In BAT, the expression of ChREBPβ and other ChREBP targets was not increased by fructose feeding in the Cre ^–^ mice ( Figure 2J and Supplementary Figure S2F,G), indicating that ChREBP activity was not induced by the dietary feeding regimen. To address, whether fructose can activate ChREBP in brown adipocytes, we incubated primary brown adipocytes with either 25 mM glucose or 1 mM fructose. The concentrations chosen reflect high postprandial glucose and fructose plasma levels in mice [ 23, 65, 66] . As predicted, glucose supplementation elevated the expression of the ChREBP targets *Acaca* and *Fasn* ( Figure 2K,L). In contrast, fructose had no effect on the ChREBP targets ( Figure 2K,L). This indicates that ChREBP activity in BAT compared to liver and other metabolically active organs is insensitive to diet-induced fructose overload, despite the ability to take up fructose.

The metabolism of fructose is mainly catalyzed by ketohexokinase (KHK, also known as fructokinase [67]), aldolase B (ALDOB [68]) and triokinase (TKFC [69]), the enzymes converting fructose into metabolites that can be incorporated into glycolysis ( Figure 2M). To test the capacity of brown adipocytes to perform this function, we determined protein expression of KHK, ALDOB and TKFC in BAT of ChREBPα ^fl/fl^ Ucp1 Cre ^+^ mice and control littermates housed at 22°C. All three enzymes were moderately expressed and independent of ChREBP activity ( Figure 2N). Of note, we found that at mRNA level BAT expresses predominantly the low-activity splice isoform *Khk*-a [51] and to a lower degree also the more active isoform *Khk-c* [51] that is typical for enterocytes and hepatocytes ( Figure 2O). Furthermore, *Khk-a* gene expression was slightly decreased upon ChREBPα-deficiency, whereas *Khk-c* expression was independent of ChREBP activity ( Figure 2O). Moreover, hexokinase 2 (HK2), which can metabolize fructose to fructose-6-phosphate, however with lower efficiency due to high Km value [70], was highly expressed in BAT and reduced upon ChREBPα deficiency ( Figure 2N). In sum, brown adipocytes do express the enzymes needed to channel fructose into glycolysis at moderate levels. Of note, the intermediates produced during this conversion appear to not activate ChREBP activity in BAT ( Figure 2J and Supplementary Figure S2F,G).

### High-fructose feeding does not increase DNL fatty acids in BAT but causes hexosylceramide accumulation in the absence of ChREBP

**Figure 3:**
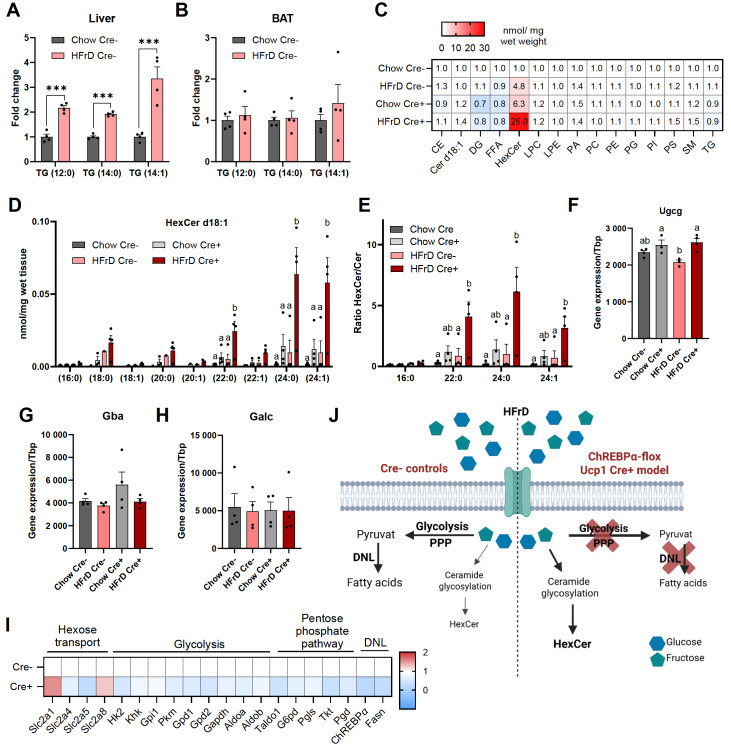
Figure 3 High-fructose feeding does not increase DNL fatty acids in BAT but causes hexosylceramide accumulation in the absence of ChREBP Fold change in lipid composition of the triglyceride (TG) fatty acids 12:0, 14:0 and 14:1 in (A) liver and (B) BAT of ChREBP-flox Ucp1 Cre– control mice fed with chow or high fructose diet. (C) Fatty acid quantity of each lipid class in BAT of ChREBPαfl/fl Ucp1 Cre+ mice and Cre– controls fed with chow or high fructose diet. n = 4, mean fold change. (D) Hexosylceramide (HexCer) fatty acid quantity in BAT of ChREBPαfl/fl Ucp1 Cre+ mice and Cre– controls fed with chow or high fructose diet. (E) Ratio between HexCer and Cer amount in BAT of ChREBPαfl/fl Ucp1 Cre+ mice and Cre– controls fed with chow or high fructose diet. Gene expressions of (F) Ugcg, (G) Gba and (H) Galc in BAT of ChREBPαfl/fl Ucp1 Cre+ mice and Cre– controls fed with chow or high fructose diet. (I) Fold change of gene expressions of carbohydrate-metabolism enzymes in BAT of ChREBPαfl/fl Ucp1 Cre+ vs Cre– mice housed at 22°C. n = 4, mean fold change. (J) Graphical summary of the proposed fructose metabolism reprogramming in ChREBP-deficient BAT after HFrD feeding. Glucose molecule is displayed as a green hexagon, and fructose molecule is displayed as blue pentagon. Data are presented as the mean ± SEM. (A,B) Student’s t-test, ***P < 0.001; (D–H) one-way-Anova, P < 0.05 is considered significant, different letters denote significant differences.

To study the impact of HFrD feeding and ChREBPα deficiency on DNL in more depth, we performed lipidomics analysis of BAT and liver samples, employing a mass spectrometry-based method that quantifies the fatty acid composition in 16 different lipid classes [ 52, 53] . In livers of Cre ^–^ mice fed with HFrD, the DNL-derived fatty acids lauric (12:0), myristic (14:0) and myristoleic acid (14:1) in the triglyceride (TG) class were strongly increased compared to chow-fed mice ( Figure 3A), suggesting a marked increase in DNL. In contrast, BAT of Cre ^–^ mice showed no induction of these TG species ( Figure 3B and Supplementary Table S1). The difference in DNL-derived fatty acid induction between liver and BAT in response to HFrD feeding ( Figure 3A,B) is unlikely to be explained by uptake of these fatty acids from the diet, as BAT takes up a higher proportion of dietary fatty acids than the liver when calculated per weight [29]. In summary, these data support the gene expression-based assumption ( Figure 2J) that, unlike in the liver, DNL in BAT is not induced by HFrD feeding.

Next, we assessed whether fructose overfeeding and ChREBPα deficiency have effects on the overall lipid balance in BAT by comparing lipid classes ( Figure 3C). The most striking change was observed for HexCer, a collective measure for glucosylceramides and galactosylceramides, isobaric sugar epimers that cannot be distinguished by our method. In Cre ^–^ mice, HFrD feeding by itself led to a trend for higher HexCer levels ( *P* = 0.37) and in Cre ^+^ mice on chow diet, a similar trend ( *P* = 0.17) for upregulation was detected ( Figure 3C). HFrD and ChREBPα deficiency in combination had an additive effect on HexCer levels, leading to an almost 30-fold increase ( *P* = 0.015) in BAT ( Figure 3C). The effects of BAT ChREBPα deficiency and fructose feeding on HexCer were not observed in liver samples ( Supplementary Figure S3A), demonstrating tissue specificity of the effect. HexCer are the precursors for gangliosides, more complex glycosphingolipids forming lipid rafts in the plasma membrane [71]. Consistent with this structural function, the majority of the HexCer in BAT contained saturated or very long-chain fatty acids (18:0, 20:0, 22:0, 24:0 and 24:1) ( Figure 3D) characterized by high hydrophobicity. Of note, the ratios of HexCer to ceramides (Cer d18:1), the starting point for cellular synthesis of HexCer, were also increased in HFrD-fed Cre ^+^ mice ( Figure 3E). Together with the observation that the concentrations of Cer d18:1 species were, overall, not different between the groups ( Supplementary Figure S3B), these findings indicate that ChREBPα deficiency and HFrD feeding promote sphingolipid glycosylation in BAT but do not significantly modulate the ceramide base of sphingolipids.

Next, we explored whether gene expression might explain the observed increases in glycosphingolipids. Expression of UDP-glucose ceramide glucosyltransferase ( *Ugcg* [72]) and UDP-galactose ceramide galactosyltransferase ( *Ugt8a* [ 73, 74] ) catalyzing the glucosylation and galactosylation, respectively, of ceramide was not regulated to a major degree in BAT of ChREBPα-deficient or HFrD-fed mice ( Figure 3F and Supplementary Figure S3C). Furthermore, the expression of the lysosomal enzymes degrading HexCer, β-glucosylceramidase ( *Gba* [75]) and galactosylceramidase ( *Galc* [76]), was unchanged ( Figure 3G,H). It was thus unlikely that the profound changes in HexCer were due to alterations in the expression of enzymes involved in formation or degradation. Glucosylceramides can also be converted via lactosylceramides to GM3 gangliosides, a lipid class demonstrated to inhibit insulin signaling and play a role in inflammation-induced insulin resistance [ 71, 77] . The main enzyme in this process is the GM3 synthase (encoded by *St3gal5* [78]). Of note, *St3gal5* gene expression was increased in BAT from ChREBPα ^fl/fl^ Ucp1 Cre ^+^ mice fed with HFrD ( Supplementary Figure S3D), suggesting that GM3 gangliosides, which are unfortunately not amenable to our method, might be also induced under this condition.

Since expression of HexCer synthesis and degradation genes showed overall little regulation, we considered ChREBP-dependent alterations in carbohydrate handling [79] as a possible explanation for the strong HexCer accumulation. When comparing 22°C-housed Cre ^–^ and Cre ^+^ mice, we found that upon ChREBPα deficiency, almost all enzymes facilitating carbohydrate-handling including pentose-phosphate pathway are strongly reduced ( Figure 3I), leading to a diminished capacity of brown adipocytes to metabolize carbohydrates. However, as shown in Figure 2, the fructose-converting enzymes KHK, ALDOB and TKFC are not regulated by ChREBP, which might lead to higher fructose metabolism rate upon ChREBPα deficiency. Based on this, we hypothesize that enhanced incorporation of sugars into sphingolipids is an alternative way to handle intracellular carbohydrate overload in brown adipocytes when oxidative carbohydrate metabolism is diminished in ChREBPα-deficient brown adipocytes ( Figure 3J).

## Discussion

In the present study, we addressed the role of fructose as an energy substrate and a regulatory molecule in BAT. We observe that fructose is internalized by BAT *in vivo* and by cultured brown adipocytes. However, our data indicate that feeding high amounts of fructose does not stimulate DNL in brown adipocytes *in vivo* and *in vitro*, as determined by DNL enzyme expression and the concentration of DNL-derived fatty acids, an indirect indicator for DNL [39]. However, a limitation of the current study is that we did not directly assess organ-specific DNL, *e*. *g*., by determining the incorporation of D _2_O into newly synthesized fatty acids in response to fructose feeding. DNL in BAT is tightly controlled by ChREBP [39], a carbohydrate-sensing transcription factor which is activated when intermediates of glycolysis and the pentose phosphate pathway are elevated [80]. Given the high amount of fructose in the diet, one can assume that a fraction of fructose is directly reaching BAT via the circulation. However, the plasma concentration of fructose that we observed was lower than that of glucose. Although we found that brown adipocytes express moderate levels of the fructose-converting enzymes KHK-C, ALDOB and TKFC, the metabolism of fructose in BAT is apparently not strong enough to stimulate ChREBP. Additionally, the lack of ChREBP activation in BAT upon HFrD feeding could be explained by the fact that the fraction of orally applied fructose that reaches the systemic circulation as glucose after conversion in enterocytes and hepatocytes [ 9, 18] is too small. Hence, the postprandial rise in systemic glucose and insulin after oral fructose administration is moderate as we and others reported [ 81– 83] . Accordingly, uptake of fructose-derived glucose into brown adipocytes via the insulin-dependent glucose transporter SLC2A4 [49] is not sufficient to activate DNL in the postprandial state in BAT. A potential confounder of our study is the use of the transgenic Ucp1-Cre ^Evdr^ mice [47] for generating the ChREBPα ^fl/fl^ Ucp1-Cre mice. Although widely used in the research field and faithfully knocking out floxed genes in brown adipocytes, several side effects of this transgene, even in hemizygous mice like the ones we used, were reported for this Cre driver recently. These include altered gene expression in BAT [84], elevated thermogenesis [84] and ectopic Cre recombinase action outside brown and beige adipocytes [ 85, 86] . Despite these confounders, it is not likely that potential side effects by the transgene affect the interpretation of our experiments in a major way, in particular because RNA-seq analysis of BAT from hemizygous Ucp1-Cre ^Evdr^ mice showed unaltered metabolic gene expression [84]. Furthermore, we observed no apparent effect on thermogenesis, another potential side effect, in the ChREBPα ^fl/fl^ Ucp1-Cre mice. Nevertheless, unwanted side effects of the transgene cannot be ruled out and a newly developed, knockin-based Ucp1-Cre line [87] or tamoxifen-inducible Ucp1-CreERT2 mice [88] may be a better choice for future experiments.

Recent studies found that *Slc2a5* expression in murine BAT is ChREBP-dependent [89], as we did, or correlates with metabolic states characterized by high ChREBP activity such as mild thermogenic activation [38] and glucose feeding [90]. However, none of these studies functionally tested the role of SLC2A5 as done in the present study. Unexpectedly, we found that the virtual loss of SLC2A5 in ChREBPα ^fl/fl^ Ucp1 Cre ^+^ mice had no effect on the uptake of radiolabelled fructose, indicating that SLC2A5 is not essential for fructose transport in BAT, an observation that is also in line with the fact that the Km value for fructose of SLC2A5 is above reported systemic fructose concentrations [91]. As an alternative, we found that Slc2a8 was the most highly expressed fructose transporter in BAT and primary brown adipocytes and notably Slc2a8 expression correlated with fructose uptake in BAT. Thus SLC2A8 might be a functional transporter for fructose in BAT, as previously proposed for liver [15]. However, it needs to be taken into account that SLC2A8 is at least predominantly localized to internal membranes [92] and may act indirectly on fructose transport through regulating *Slc2a12*, as shown in enterocytes [57]. SLC2A9 and SLC2A12 are other candidates for fructose transport in BAT. SLC2A9 is primarily a urate transporter [93] and may not transport fructose efficiently [ 93, 94] . SLC2A12 can also transport fructose albeit less efficiently than glucose [95]. Taken together, we provide evidence that *Slc2a8*, and potentially also *Slc2a9* and *Slc2a12*, may be important for fructose metabolism by brown adipocytes. However, more work needs to be done to identify the transporter responsible for fructose uptake into brown adipocytes.

An important finding of the present study was the accumulation of HexCer, encompassing galactosylceramides and glucosylceramides, in BAT of ChREBPα-deficient or fructose-fed mice, an effect that was potentiated when both conditions were combined. These lipids are mainly located in plasma membranes and the starting points for the synthesis of more complex glycosphingolipids [ 41– 43] . Of note, enhanced formation of glucosylceramides induces insulin resistance in adipocytes [96]. Strong candidates for mediating this antagonism of insulin signaling are the GM3 gangliosides that are synthesized from glucosylceramides in two steps [78]. GM3 gangliosides have previously been shown to correlate with insulin resistance in adipose tissues and cultured adipocytes [ 45, 71] . In detail, GM3 gangliosides were shown to interfere with insulin signaling by disturbing the interaction of insulin receptor with caveolin [97]. Importantly, genetic inactivation of GM3 synthase was demonstrated to revert insulin resistance [77]. Thus, the marked induction of HexCer in states of disturbed carbohydrate metabolism has the potential to induce insulin resistance, a hypothesis that can be tested in future studies employing interventions such as more prolonged fructose feeding in combination with high fat feeding.

One question that arises from our data is how ChREBPα deficiency causes HexCer accumulation. It is likely that the markedly reduced expression of glycolytic and pentose phosphate pathway enzymes prevents oxidative metabolism of glucose, diverting it into the pathway towards glucose-1-phosphate that reacts to UDP-glucose, the substrate for UDP-glucose ceramide glucosyltransferase. A more puzzling observation is that fructose feeding alone or in combination with ChREBPα deficiency strongly increases HexCer levels. Neither fructosylceramides nor UDP-fructose has been described. Furthermore, a phosphohexosemutase catalyzing the reaction of fructose-1-phosphate to glucose-1-phosphate is unknown. Thus, fructose is unlikely to feed HexCer synthesis simply by providing hexose. Rather it is tempting to speculate that fructose or a fructose metabolite stimulates HexCer through allosteric action, as described for glucokinase regulatory protein and glycogen phosphorylase [ 98, 99] .

Taken together we showed that BAT regulates fructose uptake and metabolism largely different from the liver and gut. Beyond that we provided evidence that brown adipocytes have a second mechanism next to canonical carbohydrate metabolism to handle carbohydrate surplus by incorporating sugars into HexCer. This alternative pathway is predominantly taking place in combination of failed ChREBP-dependent glucose-metabolism and fructose-rich diet and might be a mechanism to protect brown adipocyte function.

## Supporting information

25594Table_S1

25594Figure_S1

25594Figure_S2

25594Figures

25594Figure_S3

## Supplementary Data

Supplementary data are available at *Acta Biochimica et Biophysica Sinica* online.
