# Supplementary material for: Fructose uptake by brown adipose tissue is independent of carbohydrate response element-binding protein and does not cause elevated de novo lipogenesis : Fructose uptake by BAT is independent of ChREBP
Source: Acta Biochim Biophys Sin (Shanghai). 2025 Dec 18;58(7):1653–66. doi: 10.3724/abbs.2025229 (PMC13449002; doi:10.3724/abbs.2025229)

Figure 1: The ChREBP target Slc2a5 (GLUT5) is not an essential fructose transporter in brown adipose tissue.

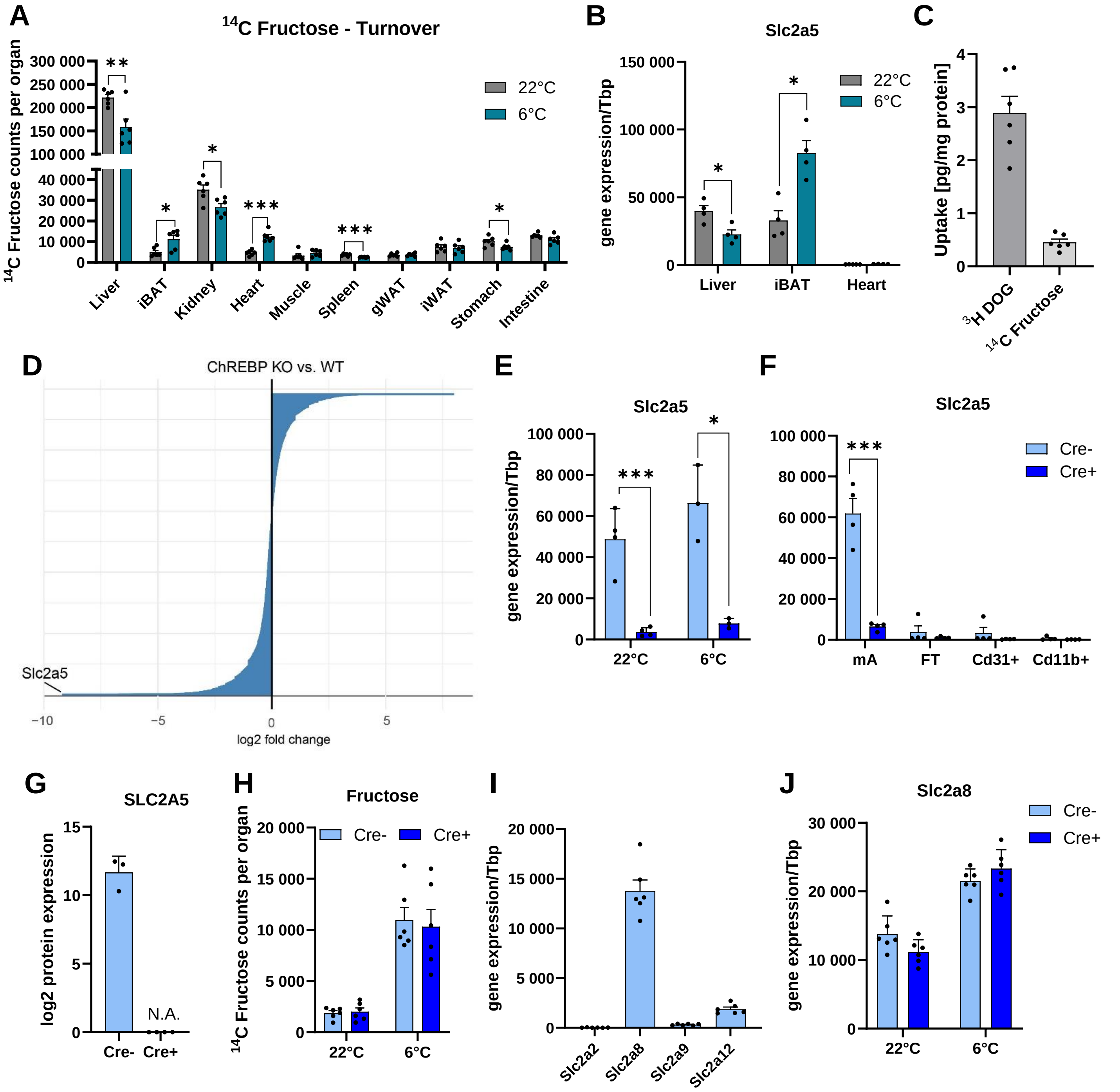

Figure 2: High-fructose feeding does not enhance DNL in BAT.

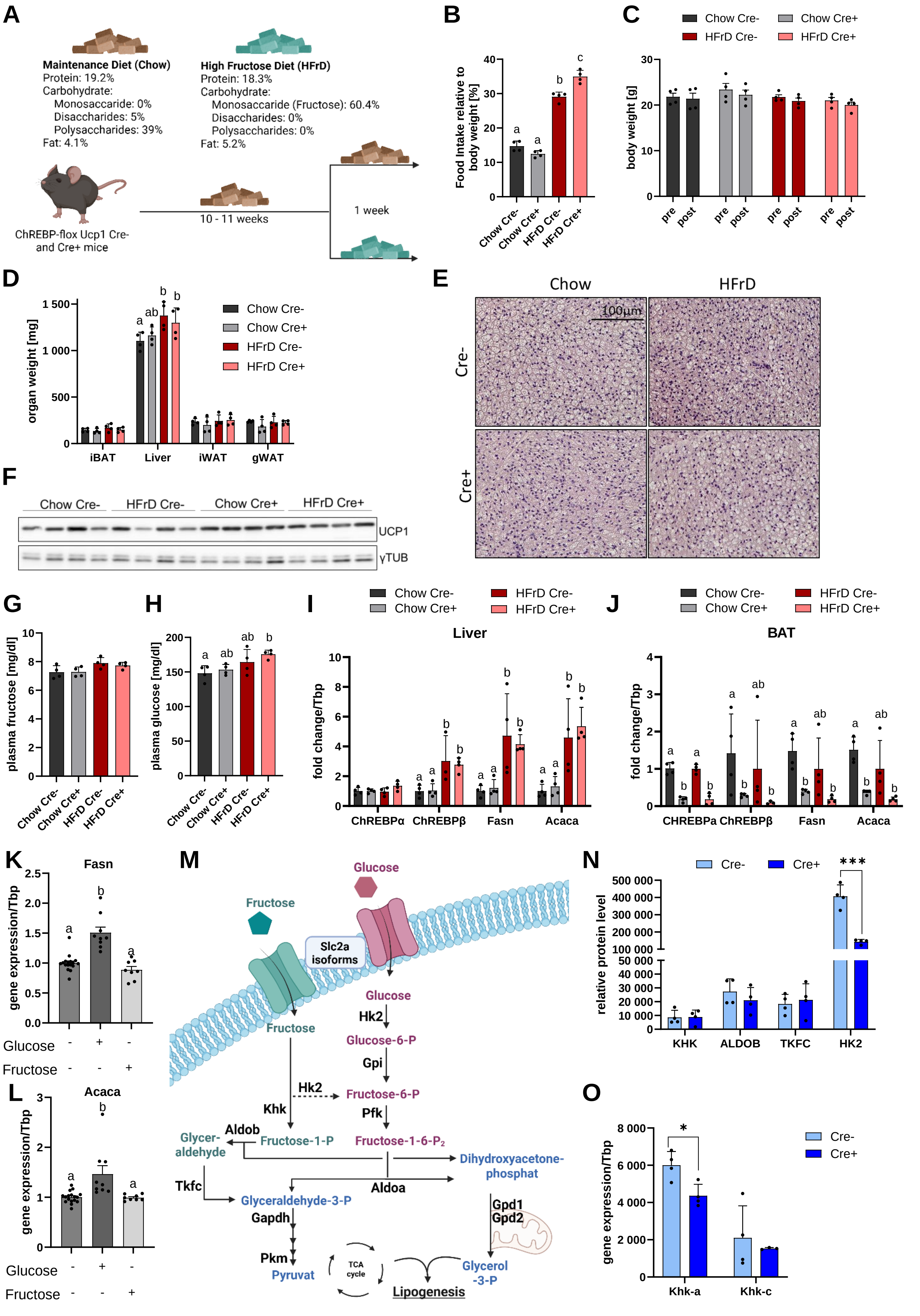

Figure 3: High-fructose feeding does not increase DNL fatty acids in BAT but causes hexosylceramide accumulation in the absence of ChREBP.

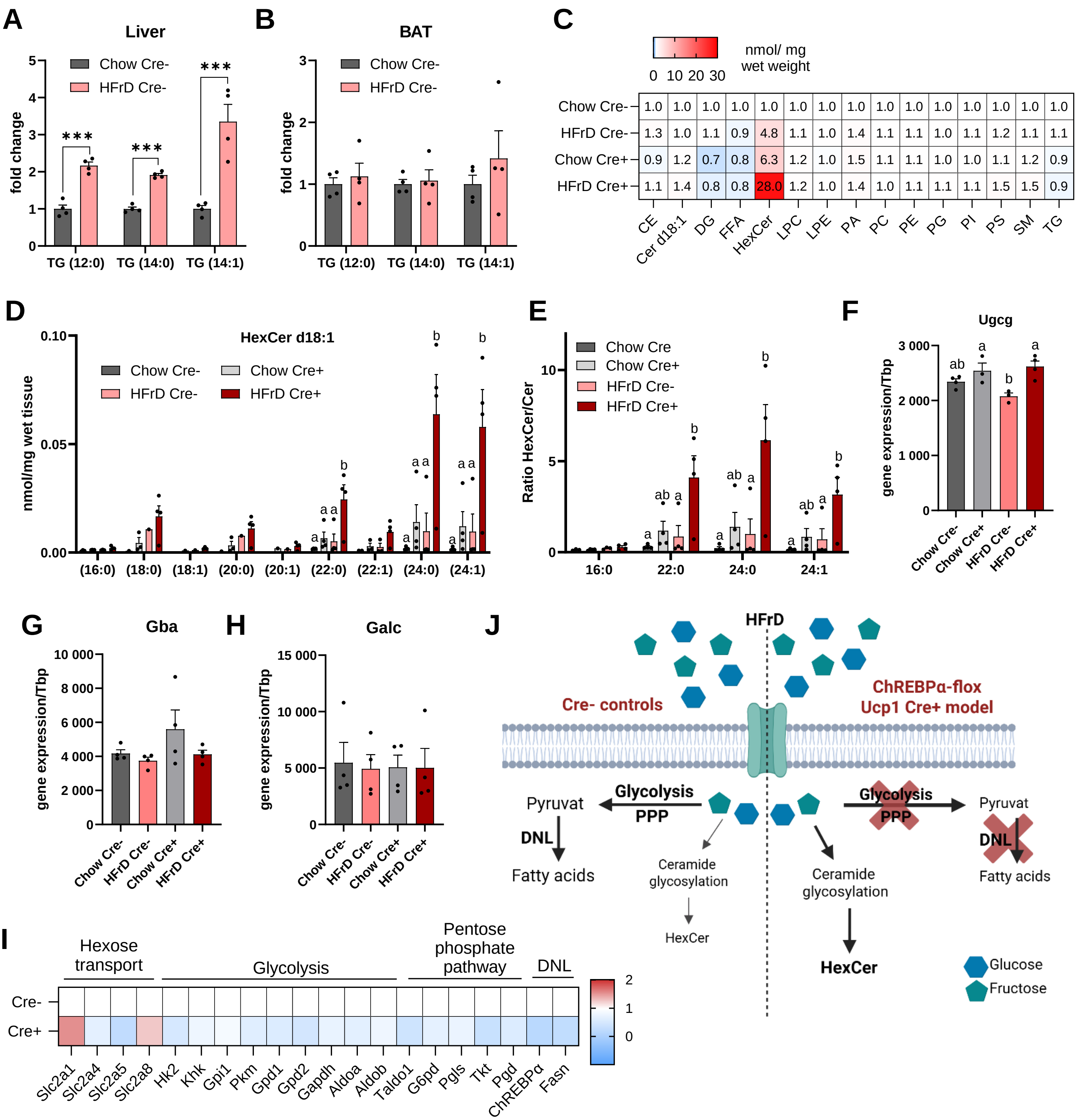

Supplement: Supplementary file 1 [file 25594Figures.pdf]
