# Supplementary material for: Fructose uptake by brown adipose tissue is independent of carbohydrate response element-binding protein and does not cause elevated de novo lipogenesis : Fructose uptake by BAT is independent of ChREBP
Source: Acta Biochim Biophys Sin (Shanghai). 2025 Dec 18;58(7):1653–66. doi: 10.3724/abbs.2025229 (PMC13449002; doi:10.3724/abbs.2025229)
Supplement: Supplementary file 2 [file 25594Figure_S1.docx]

**A**

**B**

**D**

**Supplementary Figure S1. Deoxyglucose disposal, fructose transporter expression and DNL in BAT** (A) ^3^H deoxyglucose (DOG) counts per organ measured in organs of male wild-type mice housed at 22°C or 6°C for 7 days. (B) Fold change in lipid composition of the triglyceride (TG) fatty acids 12:0, 14:0, 14:1, 16:0 and 16:1 in BAT of ChREBPα^fl/fl^ Ucp1 Cre^+^ mice and Cre^−^ controls. (C) Gene expression of *Slc2* isoforms in primary brown adipocytes. *n* = 8 from independent experiments. (D−F) Correlation of ^14^C fructose counts per organ with (D) *Slc2a8,* (E) *Slc2a9 or* (F) *Slc2a12* copy numbers measured in BAT of Cre- mice. Data are presented as the mean ± SEM, Student’s *t*-test. **P* < 0.05, ***P* < 0.01, ****P* < 0.001.

**E**

**F**

**C**
