# Supplementary material for: Fructose uptake by brown adipose tissue is independent of carbohydrate response element-binding protein and does not cause elevated de novo lipogenesis : Fructose uptake by BAT is independent of ChREBP
Source: Acta Biochim Biophys Sin (Shanghai). 2025 Dec 18;58(7):1653–66. doi: 10.3724/abbs.2025229 (PMC13449002; doi:10.3724/abbs.2025229)
Supplement: Supplementary file 3 [file 25594Figure_S2.docx]

**F**

**A**

**B**

**C**

**D**

**E**

**Supplementary Figure S2. UCP1 expression, plasma parameters and DNL enzyme expression in response to high fructose feeding and ChREBP deficiency** (A) Quantification of the UCP1 western blot from iBAT of ChREBPα^fl/fl^ Ucp1 Cre^+^ mice and Cre^−^ controls fed with chow or high fructose diet shown in **Figure 2F**. Plasma (B) triglyceride, (C) cholesterol, (D) insulin and (E) ketone bodies of ChREBPα^fl/fl^ Ucp1 Cre^+^ mice and Cre^−^ controls fed with chow or high fructose diet. (F) Western blot analysis of ACC and FASN from iBAT and liver of ChREBPα^fl/fl^ Ucp1 Cre^+^ mice and Cre^−^ controls fed with chow or high fructose diet. (G) Gene expressions of *Acly*, *Scd1* and *Elovl6* in BAT of ChREBPα^fl/fl^ Ucp1 Cre^+^ mice and Cre^−^ controls fed with chow or high fructose diet. Data are presented as the mean ± SEM, one-way-Anova. *P* < 0.05 is considered significant, different letters denote significant differences.

**G**
