# Supplementary material for: Fructose uptake by brown adipose tissue is independent of carbohydrate response element-binding protein and does not cause elevated de novo lipogenesis : Fructose uptake by BAT is independent of ChREBP
Source: Acta Biochim Biophys Sin (Shanghai). 2025 Dec 18;58(7):1653–66. doi: 10.3724/abbs.2025229 (PMC13449002; doi:10.3724/abbs.2025229)
Supplement: Supplementary file 4 [file 25594Figure_S3.docx]

Figure S3

**A**

**B**

**C**

**D**

**Supplementary Figure S3.** **Lipidome analysis and sphingolipid gene expression in BAT from high fructose-fed mice** (A) Fatty acid quantity of each lipid class in liver of ChREBPα^fl/fl^ Ucp1 Cre^+^ mice and Cre^−^ controls fed with chow or high fructose diet. *n* = 4, mean fold change. (B) Ceramide d18:1 (Cer d18:1) fatty acid quantity in BAT of ChREBPα^fl/fl^ Ucp1 Cre^+^ mice and Cre^−^ controls fed with chow or high fructose diet. (C,D) Gene expressions of *Ugt8a* (C) and *St3gal5* (D) in BAT of ChREBPα^fl/fl^ Ucp1 Cre^+^ mice and Cre^−^ controls fed with chow or high fructose diet. Data are presented as the mean ± SEM, one-way-Anova. *P* < 0.05 is considered significant, different letters denote significant differences.
