# Supplementary material for: Fructose uptake by brown adipose tissue is independent of carbohydrate response element-binding protein and does not cause elevated de novo lipogenesis : Fructose uptake by BAT is independent of ChREBP
Source: Acta Biochim Biophys Sin (Shanghai). 2025 Dec 18;58(7):1653–66. doi: 10.3724/abbs.2025229 (PMC13449002; doi:10.3724/abbs.2025229)
Supplement: Supplementary file 5 [file 25594Table_S1.docx]

**Supplementary Table S1.** ***P* values calculated by Student’s *t*-test Chow vs HFrD** Fatty acids were measured in BAT of ChREBPα^fl/fl^ Ucp1-Cre- mice fed with chow diet or HFrD
